# Central Nervous System Involvement in Acute Myeloid Leukemia: From Pathophysiology to Neuroradiologic Features and the Emerging Role of Artificial Intelligence

**DOI:** 10.3390/jcm15031187

**Published:** 2026-02-03

**Authors:** Rafail C. Christodoulou, Rafael Pitsillos, Vasileia Petrou, Maria Daniela Sarquis, Platon S. Papageorgiou, Elena E. Solomou

**Affiliations:** 1 Department of Radiology, Stanford University School of Medicine, Stanford, CA 94305, USA; 2 Neurophysiology Department, Cyprus Institute of Neurology and Genetics, Nicosia 2371, Cyprus; rafaelp@cing.ac.cy; 3 Department of Medicine, University of Ioannina, 45110 Ioannina, Greece; md07010@uoi.gr; 4 Department of Medicine, Universidad de Carabobo, Valencia 2001, Venezuela; mdsarquis58@gmail.com; 5 Department of Medicine, National and Kapodistrian University of Athens, 15772 Athens, Greece; platopap@med.uoa.gr; 6 Department of Internal Medicine-Hematology, University of Patras Medical School, 26500 Rion, Greece

**Keywords:** acute myeloid leukemia, central nervous system, MRI, neuroimaging, myeloid sarcoma, leptomeningeal disease, artificial intelligence, radiomics, machine learning

## Abstract

**Background/Objectives**: Central nervous system (CNS) involvement in acute myeloid leukemia (AML) is a rare but important complication linked to poor outcomes. Diagnosing it is difficult because neurological symptoms are often subtle or nonspecific, and conventional cytology and imaging have limitations. This review summarizes current evidence on the neuroradiologic features of CNS infiltration in AML and explores the growing role of artificial intelligence (AI) in enhancing detection and characterization. **Methods**: A thorough narrative review was conducted using PubMed, Scopus, and Embase, employing key terms related to AML, CNS involvement, MRI, CT, PET, AI, machine learning, deep learning, and radiomics. Of several thousand records, 138 relevant studies were selected and analyzed across four main areas: neuroradiologic patterns, imaging biomarkers, AI and radiomics applications, and emerging computational trends. **Results**: Imaging findings in AML mainly include myeloid sarcomas (isointense on T1, hyperintense on T2/FLAIR, restricted diffusion) and leptomeningeal enhancement. Secondary ischemic or hemorrhagic lesions may indicate brain leukocytosis. MRI proved more sensitive than CT, while PET/CT helped detect extramedullary disease. Recent AI and radiomics models showed high tumor classification and prognosis accuracy in similar CNS conditions, indicating significant potential for application in AML-CNS. **Conclusions**: Combining AI-based image analysis with multimodal neuroimaging could significantly improve diagnostic accuracy and personalized treatment for CNS involvement in AML. Progress is still challenged by the rarity of the condition and the lack of large, annotated datasets.

## 1. Introduction

AML is an aggressive hematologic malignancy marked by rapid progression, high relapse rates, and substantial mortality despite advances in molecular classification and targeted therapies. Although contemporary genomic profiling has refined risk stratification and treatment selection, AML remains clinically challenging because of its biological heterogeneity, frequent treatment resistance, and the emergence of extramedullary disease [1,2,3]. Among these, CNS involvement is a particularly critical yet underrecognized complication, associated with significant neurological morbidity, therapeutic limitations, and poor prognosis. AML arises from the clonal expansion of myeloid progenitor cells derived from pluripotent hematopoietic stem cells (HSCs), which acquire multiple genetic mutations and epigenetic modifications. These aberrations confer proliferative and survival advantages while impairing normal differentiation, ultimately leading to the accumulation of malignant myeloid clones and the development of myeloid neoplasms [1,2]. Consequently, the hematopoietic process is disrupted as leukemic proliferation within the bone marrow interferes with normal blood cell formation [4]. Given that the molecular alterations are crucial to leukemogenesis, recent advances in genomic profiling have enabled the genetic classification of AML based on recurrent mutations in genes regulating cell survival, signal transduction, and RNA splicing [4,5]. This genomic framework was adopted by the latest World Health Organization (WHO), Society for Hematopathology, and the European Association for Hematopathology classifications, and provides a biologically driven approach to diagnosis and prognosis, emphasizing the clinical relevance of mutations such as NPM1, FLT3, and TP53 [3,6,7,8].

AML represents one of the most common forms of acute leukemia, with an estimated incidence of approximately 4 cases per 100,000 individuals per year. Despite its relative frequency, AML is associated with a poor overall survival rate, mainly due to its heterogeneous biology and often subtle or nonspecific clinical presentation that delays diagnosis and treatment initiation [9,10]. Aside from hematologic and nonspecific manifestations such as anemia, fatigue, and hemorrhagic events, AML rarely involves the central nervous system (CNS), with an incidence of 1% among new AML cases and higher rates in pediatric AML [11]. CNS infiltration may occur as parenchymal lesions within the brain or, more commonly, as leptomeningeal involvement [11,12].

Regarding CNS involvement in AML, Alakel N. et al. reported a higher incidence among patients with adverse cytogenetic profiles, elevated serum lactate dehydrogenase (LDH) levels, and concurrent FLT3 and NPM1 mutations in 2017. These patients exhibit a broad spectrum of neurological manifestations, ranging from mild symptoms such as headache and cranial nerve palsies to severe complications including intracerebral hemorrhage, elevated intracranial pressure, and seizures. CNS involvement in AML usually indicates severe lab findings, advanced disease, high leukocyte counts, monocytic subtypes, and poor prognosis [12,13].

Clinical manifestations of CNS involvement in AML are rare, and several challenges limit both diagnosis and treatment, ultimately reducing survival. These can be grouped into two categories: barriers to early and accurate detection, and restricted therapeutic options. Although flow cytometry (FCM) has demonstrated improved detection sensitivity and accuracy compared to conventional cytology [14], both techniques remain limited in reliably identifying CNS involvement. Another important consideration is the invasive nature of lumbar puncture (LP) and the potential adverse effects that may occur following the procedure. Despite evidence that CSF cytometry can detect blasts in approximately 40% of asymptomatic AML patients, LP remains underutilized, particularly in this population, due to its invasiveness [15,16]. These limitations underscore the need for advanced imaging biomarkers that enable neuroradiologists to detect CNS disease in high-risk patients, even in the absence of overt symptoms [17,18]. Neuroimaging is therefore essential, with magnetic resonance imaging (MRI) as the primary modality for lesion detection and characterization, complemented by positron emission tomography (PET) scans [12]. Identifying specific imaging biomarkers improves diagnosis and allows precise disease monitoring, addressing the limitations in treatment options. Current therapies remain limited in preventing relapse, with intrathecal approaches representing a promising but still developing strategy [19].

As previously indicated, neuroimaging is crucial in detecting CNS infiltration in AML, as most patients with neurological manifestations present at advanced stages. Lesions on T1- or T2-weighted MRI sequences may be subtle, and meningeal involvement may be minimal, making conventional MRI relatively low in sensitivity [20]. Artificial intelligence (AI), including machine learning (ML) and deep learning (DL), has the potential to overcome these limitations by detecting and characterizing subtle imaging changes. By leveraging large-scale imaging datasets, AI models can recognize complex spatial patterns, integrate multimodal data (such as MRI and PET), and generate predictive biomarkers for disease progression and treatment response. These can complement traditional approaches and assist neuroradiologists in making critical diagnostic and clinical decisions [21]. Hence, this narrative review aims to elucidate the underlying pathophysiology and risk factors contributing to CNS involvement in AML, while highlighting the diagnostic and prognostic value of neuroimaging. Furthermore, it explores the emerging role of AI in refining disease detection, monitoring progression, and evaluating therapeutic efficacy in CNS disease in AML. This relatively novel topic has not been extensively addressed in previous studies. Our review is among the first to integrate this pathology’s pathophysiology and radiological aspects with the emerging contributions of AI in the field.

## 2. Results

### 2.1. Literature Overview

The 138 studies included span publications from 2000 to 2025, reflecting ongoing advancements in neuroimaging and computational modeling. Early studies primarily reported radiologic case series of CNS infiltration in AML, whereas recent research emphasizes AI-driven image analysis and multimodal data fusion for more precise diagnostics. 3.

### 2.2. Neuroradiologic Features of CNS Involvement

Neuroimaging in AML reveals diverse patterns depending on the infiltration route and extent:

- Myeloid sarcomas (chloromas) are iso- to hypointense on T1 MRI and hyperintense on T2/FLAIR, often showing homogeneous contrast enhancement and restricted diffusion on ADC maps. Dural-based lesions may resemble meningiomas but usually lack calcifications and can cause local bone destruction.
- Leptomeningeal disease: Presents as diffuse or nodular meningeal enhancement on post-contrast T1 WI, sometimes extending to cranial or spinal nerves, ependymal surfaces, or tentorium.
- Indirect CNS involvement: Leukostasis-related infarcts and microbleeds appear as diffusion-restricted foci and low-signal blooming artifacts on SWI.
- MRI is the most sensitive modality, especially for subtle parenchymal or meningeal disease, while CT is useful for detecting hemorrhagic or mass-effect issues. PET/CT offers additional value in identifying extramedullary sites and assessing treatment response.

### 2.3. Artificial Intelligence and Radiomics

The review shows a growing use of AI and radiomics in CNS imaging over the past ten years (Table 1).

- Deep learning models like AML-Net and similar CNN architectures have achieved high accuracy in brain tumor segmentation, offering adaptable frameworks for detecting myeloid sarcomas.
- Radiomic analyses using MRI texture, intensity, and shape features have reached AUCs between 0.82 and 0.97 for lesion classification and survival prediction in related diseases.
- Transfer learning and federated AI are promising approaches to address data limitations, enabling pretrained models trained on large datasets (e.g., BraTS, UCSF-PDGM, TCIA) to be adapted for rare AML-associated CNS manifestations.

### 2.4. Summary of Key Insights

**Figure 1:**
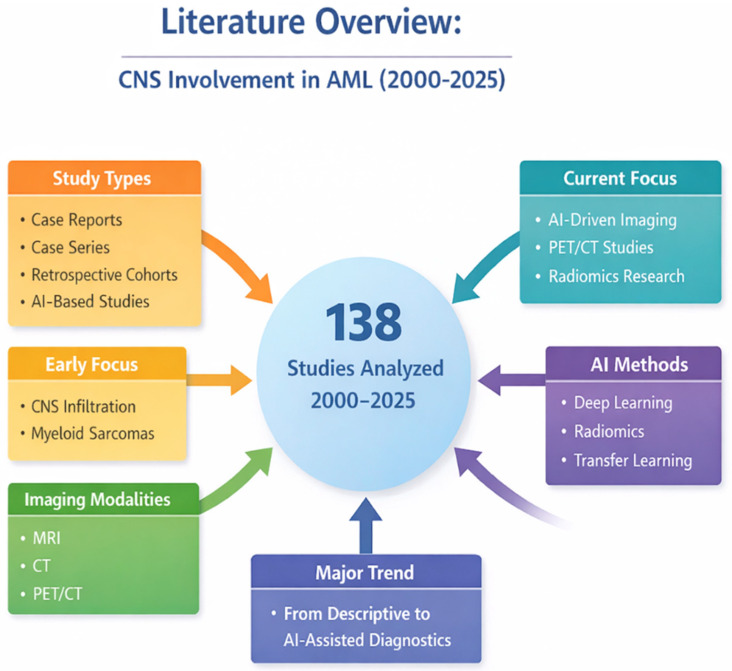
Overview of the literature on CNS involvement in AML (2000–2025). The diagram summarizes the study types, imaging methods, and analysis approaches across 138 studies, showing a shift from descriptive imaging to AI-assisted neuroimaging, including deep learning, radiomics, and transfer learning. Created in BioRender. Christodoulou, R. (2026) Available online: https://BioRender.com/3jubwzb (accessed on 20 December 2025).

Overall, the current literature highlights that CNS involvement in AML, though uncommon, has distinct imaging features that can aid diagnosis and management (Figure 1).

AI-assisted image analysis, radiomics, and multimodal integration are emerging tools that can improve detection sensitivity, characterize disease heterogeneity, and support personalized therapy. However, the main obstacle to clinical application is the shortage of large, disease-specific imaging databases for validation.

## 3. Discussion

### 3.1. Pathophysiology and Mechanisms of CNS Infiltration

**Figure 2:**
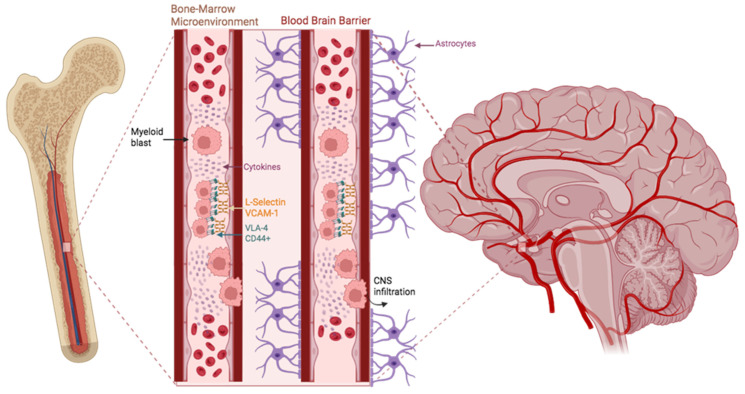
CNS infiltration of AML blasts. Malignant myeloid progenitors escape the bone marrow microenvironment through cytokine release and altered expression of cell adhesion molecules, subsequently crossing the blood–brain barrier to infiltrate the meninges or the brain parenchyma. Created in BioRender. Christodoulou, R. (2026) Available online: https://BioRender.com/fii6xvy (accessed on 20 December 2025).

Before investigating the CNS disease in AML, it is essential to elucidate the underlying pathophysiology and key molecular mechanisms driving leukemic blast migration and escape from the bone marrow (BM) microenvironment. Initially, normal hematopoietic stem cells (HSCs) in the BM acquire genetic mutations and molecular alterations that enable uncontrolled proliferation and confer resistance to apoptosis, forming a single myeloid progenitor cell clone lineage [15,31]. These mutations are subsequently compounded by additional somatic driver alterations, such as Nucleophosmin 1 (NPM1) and FMS-like Tyrosine Kinase 3 (FLT3) mutations, ultimately leading to leukemogenesis and the acquisition of hallmark tumor characteristics, as extensively described in the literature [6,7,32]. These blasts multiply indefinitely, occupying the BM and causing a marked decline in the population of normal hematopoietic precursor cells [33]. AML cells egress consequently from the blood–marrow barrier, a mechanical “domain” composed of the BM endothelial cells, along with stromal and mesenchymal cells that prevent re-entry of mature erythrocytes and platelets [34,35], through the involvement of multiple chemotactic and adhesive factors and complex signaling pathways [15]. The most significant molecules described in the literature as being expressed in AML blasts include the integrins VLA-4 and VLA-5, and LFA-1 that binds to ICAM-1 for leukocyte migration; the transmembrane glycoprotein molecules L-selectin and VCAM-1, which regulate leukocyte adhesion and mobilization; and several clusters of differentiation such as CD34, CD44, and CD56 [36,37,38,39,40,41]. For instance, CD56, a natural killer (NK) cell adhesion molecule dysregulated in approximately 20% of AML cases, has recently been characterized as a significant prognostic marker due to its involvement in the MAPK pathway and its association with chemoresistance in CD56^+^ leukemic blasts [41,42]. Figure 2 depicts AML cells escaping from the BM microenvironment via the blood sinuses (marrow capillaries) and eventually migrating into extramedullary tissues [43].

Upon dissemination of AML cells into the circulation, the CNS is a rare yet possible site of extramedullary infiltration. As in most myeloid leukemias, CNS infiltration in AML primarily occurs via hematogenous dissemination through the “leaky” blood–brain barrier (BBB). The permeability of the BBB is regionally heterogeneous [44]; for instance, the choroid plexus displays increased endothelial fenestrations. AML cells therefore migrate into the CNS circulation and traverse the BBB through a multistep process that begins with adhesion, followed by the release of chemokines and cytokines that widen tight junctions, and ultimately causes extracellular matrix disruption mediated by metalloprotease activity [45]. AML blasts migrate toward the brain after inducing a hypoxic, highly angiogenic microenvironment within the CNS perivascular spaces, thereby facilitating their survival and further infiltration [36,46]. In addition to hematogenous spread, lymphatic dissemination may contribute to CNS invasion in AML. The discovery of the glymphatic system has challenged the long-standing view of the CNS as an immune-privileged site [47], a perivascular network that facilitates the clearance of metabolic waste and functions analogously to the peripheral lymphatic system- prompting a re-evaluation of CNS immune isolation [48,49]. When glymphatic flow is impaired, these interconnected pathways may enable the retrograde migration of malignant cells within the CSF, ultimately promoting their entry into the brain parenchyma [15]. As a consequence of CNS metastasis in AML, infiltration may manifest as leptomeningeal seeding involving the arachnoid and pia mater [50], parenchymal (intracerebral) infiltration typically presenting as chloromas or granulocytic sarcomas [51], or, more rarely, as dural or epidural involvement [52]. In addition to the inherent biology of leukemic cells and molecular adhesion processes, new evidence indicates that systemic host factors, such as the gut microbiota, may play a role in AML development and disease progression. Changes in microbiota composition have been linked to immune system imbalance, persistent inflammation, compromised intestinal barrier function, and altered responses to chemotherapy in AML patients [53]. These systemic influences can indirectly affect endothelial permeability, immune monitoring, and inflammatory signaling, potentially impacting the likelihood of extramedullary manifestations, including CNS involvement. While CNS involvement is a common and recognized complication in ALL, it is relatively rare in AML. This disparity is mainly due to differences in cellular origin, biological characteristics, and tissue targeting, with lymphoid blasts more likely to migrate to the CNS and spread to the leptomeninges [54]. Conversely, CNS involvement in AML usually occurs in cases with high-risk molecular markers, hyperleukocytosis, or extramedullary disease, rather than from routine prophylactic spread. CNS involvement in AML is defined by the detection of leukemic blasts in CSF or by evidence of leptomeningeal or parenchymal disease on neuroimaging in the appropriate clinical context. Diagnosis typically relies on demonstrating malignant cells in CSF cytology and flow cytometry, with imaging modalities such as MRI complementing CSF analysis by identifying meningeal enhancement or focal lesions suggestive of infiltration [55].

Several risk factors have been investigated and identified as significant for CNS involvement in AML [56]. A recent study by Seki J. T. et al. (2025) [57] employed next-generation sequencing (NGS) to characterize key genetic alterations associated with CNS disease in AML. This study performed CSF analysis in 259 patients who underwent lumbar puncture, revealing CNS involvement in approximately 12% of cases. The most relevant genetic risk factors identified were NPM1 and FLT3 mutations, occurring in 48% and 29% of cases, respectively [57]. NPM1, a ubiquitously expressed nucleolar protein, has been implicated in the ARF–p53 tumor-suppressor pathway, contributing to cell-cycle regulation and genomic stability [58]. The FLT3 tyrosine kinase receptor is a major regulator of hematopoietic cell survival and differentiation, and its frequent internal tandem duplication (ITD) mutations result in aberrant protein activation [59]. The co-occurrence of NPM1 and FLT3 mutations is associated with higher rates of CNS involvement [13,57]. An increased white blood cell (WBC) count has also been strongly associated with CNS disease in AML. Patients with CNS-positive AML are more frequently found to have WBC counts exceeding 50–100 × 10^9^/L [60]. Moreover, elevated WBC levels have been linked to FLT3-ITD mutations and CD56 positivity -two factors that are both indicative of aggressive disease biology and associated with a higher likelihood of CNS involvement [41,61]. Other significant risk factors reported in the literature include the myelomonocytic and monocytic AML subtypes (M4 and M5 according to the French–American–British [FAB] classification) [62], elevated lactate dehydrogenase (LDH) levels [63], and aberrant cytogenetic profiles. For instance, chromosome 16 inversion and trisomy 8, as demonstrated by Shihadeh F. et al. in 2012 [64], and t(8;21) translocation [65], have been associated with extramedullary tumors.

### 3.2. Clinical Manifestation of CNS Disease in AML

AML can lead to neurological impairment and nervous system manifestations through two main mechanisms: directly, via infiltration of malignant myeloid cells into the nervous system, or indirectly, as a consequence of systemic disease progression or adverse effects of treatment modalities, such as chemotherapeutic agents or immunosuppressive therapy following hematopoietic stem cell transplantation [66]. Regarding direct CNS infiltration, the most common manifestation in patients with AML is leptomeningeal disease, which arises from the seeding of AML blasts after they migrate from the BM and subsequently penetrate the CNS, as previously described [57]. Leptomeningeal disease primarily occurs via hematogenous dissemination or through regions of a compromised BBB, such as the choroid plexus, and, more rarely, through direct invasion from adjacent tissues. It manifests with subtle, nonspecific nervous system symptoms, including seizures, confusion, or headaches [50]. Cranial nerve palsies or radicular involvement of spinal nerves may also develop due to leukemic cell infiltration into the CNS, as reported by Zheng C. et al. [67]. Leukemic blasts can also metastasize directly to the brain parenchyma, forming the well-characterized and widely reported chloromas (also referred to as granulocytic sarcomas or myeloid sarcomas). Chloromas typically manifest with progressive frontal headaches, reflecting their mass effect within the affected brain regions [51,68]. Myeloid sarcomas can also arise within the spinal epidural space, resulting in compression of the spinal cord or nerve roots and manifesting as radiculopathies [52,69], as illustrated by a reported case of recurrent acute promyelocytic leukemia (APL) presenting with right leg pain [70]. When the lesion is situated below the L1–L3 vertebral levels, it may precipitate cauda equina syndrome, a neurosurgical emergency characterized by compression of the lumbosacral nerve roots, with a high risk of permanent motor and sensory deficits, including paraplegia [71,72]. In indirect CNS involvement, most events result from hematological abnormalities such as hyperleukocytosis, anemia, thrombocytopenia, and other related derangements. Transient ischemic attack and intracranial hemorrhage in patients with APL have been reported in the literature [73,74,75], syncope as a primary symptom in AML [76], or even pituitary apoplexy in rarer cases of AML, occurring with thrombocytopenia due to the disease [77]. Rare cases of diabetes insipidus due to AML have also been reported [78,79,80].

### 3.3. Neuroimaging Findings in CNS Involvement in AML

Paraclinical evaluation is crucial for detecting, characterizing, and monitoring CNS involvement in AML. Neuroimaging, such as MRI, CT, and PET, identifies leukemic infiltration and CNS complications, while cerebrospinal fluid analysis provides cytological, immunophenotypic, and molecular insights, despite limited sensitivity in asymptomatic patients [15,81]. Emerging tools such as radiomics and AI enhance lesion detection, reduce variability, and aid risk stratification. Molecular and hematologic markers, such as FLT3 and NPM1 mutations, elevated LDH, and leukocytosis, provide insights into disease aggressiveness and relapse risk [82]. Combining imaging, CSF, and molecular data advances precision diagnostics and personalized CNS disease management in AML.

The clinical manifestations of CNS involvement in AML may be subtle or nonspecific, often escaping clinical detection. Comprehensive and systemic evaluation is essential to identify occult disease features [36]. Clinical investigation for detecting nervous system invasion by myeloid leukemia blasts primarily uses neuroimaging as a non-invasive modality for tumor recognition. A 2020 retrospective cohort study reported the findings from the investigation of 84 patients with CNS disease in AML, among whom 62% demonstrated aberrant MRI features [20]. While several studies have extensively investigated CNS involvement in other hematologic malignancies, particularly acute lymphoblastic leukemia and various lymphomas, the available literature on AML remains comparatively scarce. There is a notable lack of systematic research addressing imaging characteristics of CNS infiltration in AML. Therefore, this section will be subdivided according to the various manifestations of CNS involvement and their corresponding detection strategies and imaging patterns. Table 2 summarizes the main radiologic findings of CNS disease in AML.

CNS disease in AML commonly presents as a solid mass composed of metastasized myeloid malignant cells, referred to as chloroma, granulocytic sarcoma, or myeloid sarcoma [83]. Before the advent of MRI, and still in current emergency practice, computed tomography (CT) remains the first-line imaging modality for excluding neurosurgical emergencies. Multiple small lesions are more frequently observed on brain CT than solitary larger masses [84]. These lesions typically demonstrate minimal contrast enhancement and are predominantly extracerebral, arising from the dura as focal extraaxial isodense or hyperdense soft-tissue masses rather than located within the brain parenchyma. Edema may also appear surrounding the masses [83,84]. With advances in MRI technology, malignancies can now be detected with greater accuracy, allowing neuroradiologists to characterize tumor features more precisely through multiple MRI sequences developed in recent years [20]. Chloromas appear on MRI as isointense or hypointense on T1-weighted imaging (T1WI), with homogeneous contrast enhancement, and, conversely, as hyperintense or isointense on T2-weighted imaging (T2WI). Hypointense edema also contributes to mass effect manifestations and compression of brain regions and nuclei, depending on their location [85]. Restricted diffusion (DWI) with confirmation on low-ADC maps may also be observed on MRI, reflecting increased cellularity [86,87]. Regarding chloromas arising from the extraaxial space, although dural-based cranial granulocytic sarcomas may exhibit imaging characteristics resembling meningiomas, the latter typically demonstrate calcifications rather than osseous destruction [81,85]. According to a study by Ooi et al., other common sites of granulocytic sarcoma involvement include the spinal cord, nerve roots, and the intracerebral and epidural locations. The same study also highlighted a possible enhancing rim surrounding these masses, a feature that may easily lead to misdiagnosis as an infectious or inflammatory process [88]. A recent investigation conducted in 2023 evaluated MRI findings associated with CNS involvement across various leukemia subtypes, identifying four cases of AML infiltration located within the brain parenchyma, cranial nerves, and in two instances, with mixed localization. The parenchymal lesions demonstrated isointensity on T1WI and hyperintensity on T2WI and FLAIR [80]. Studies have reported that recurrent AML presents as isolated intracranial or extra-axial masses, which are generally associated with poor outcomes [65,89]. PET imaging has also been explored for detecting myeloid sarcomas in AML, primarily in extramedullary sites outside the CNS, with promising results showing enhanced tracer uptake in meningeal or intracerebral lesions [90,91].

In a related line of investigation, CNS leukemia may also present with leptomeningeal metastasis rather than a focal solid mass [20]. This is due to CSF seeding of the blasts that eventually invade the meninges [66]. Leptomeningeal disease appears most frequently on MRI as pial surface signal enhancement on post-contrast T1WI and as multinodular lesions. Other less common MRI features include alterations in white matter and signal enhancement of cranial or spinal nerves, depending on the location [92,93]. Common sites of leptomeningeal infiltration are the ventricular ependymal surfaces, the tentorium, and the outermost cortical layer (cerebral convexity) [92]. Several studies have reported their results on leptomeningeal dissemination in AML. A study by Liu et al. described the imaging characteristics of 14 patients with CNS leukemic involvement. Among these, MRI of a patient with acute promyelocytic leukemia (APL) revealed patchy leptomeningeal enhancement in both parietal lobes. In another case of AML, multiple scattered lesions were identified within the right lateral ventricle, accompanied by heterogeneous signal intensity on diffusion-weighted imaging (DWI). Similarly, a patient with the M4 subtype of AML demonstrated meningeal enhancement and heterogeneous DWI signal intensity [94]. Meningeal involvement may also occur as a manifestation of recurrent AML. This was reported in a case of a patient who developed pain and weakness in both upper and lower extremities following complete therapeutic remission of NPM1/FLT3-ITD-positive AML, in whom MRI subsequently revealed meningeal seeding consistent with myeloid sarcoma [82]. In rare instances, leptomeningeal relapse of AML may develop following resection of intracranial myeloid sarcoma. Qian et al. (2015) reported a case in which contrast-enhanced T1-weighted MRI demonstrated leptomeningeal thickening and enhancing masses along the lumbar and sacral spinal cord [95].

CNS manifestations in AML can also present with neuroimaging findings suggesting indirect nervous system involvement. An uncommon case of AML presenting with multiple hyperdense intracerebral lesions on CT, initially misinterpreted as chloromas but later identified as hemorrhagic infarcts secondary to leukostasis (WBC > 100 × 10^9^/L), was reported by Algharras et al. [96]. Chronic blood products due to microbleed can also be detected with susceptibility-weighted imaging (SWI), where low signal and blooming artifacts are consistent with the diagnosis [87]. Similarly, MRI findings in another patient demonstrated multiple disseminated hyperintense foci on diffusion-weighted imaging (DWI) within the bilateral periventricular white matter and the left parieto-occipital junction, consistent with recent ischemic episodes [73]. Castro et al. recently reported an interesting incident of bilateral globus pallidus involvement, showing hypodense lesions on CT and hyperintense on T2-weighted/FLAIR MRI with restricted diffusion and mild enhancement, consistent with hypoxic injury [97]. Neuro-ophthalmic manifestations are rare in AML, even less frequent than in acute lymphoid leukemia. Imaging across cases revealed heterogeneous CNS and orbital involvement: CT showed orbital roof lytic lesions and soft-tissue masses, while MRI (T1 post-contrast, T2-weighted, FLAIR, and DWI) demonstrated leptomeningeal and cranial nerve enhancement—especially of the third, fifth, and sixth nerves—optic nerve sheath thickening, and cerebellar cytotoxic edema, reflecting leukemic infiltration or treatment-related gliosis [98]. Although most cases of CNS leukemia are asymptomatic, they can sometimes present with clinical symptoms that reflect the location and biological nature of leukemic infiltration. For example, leptomeningeal enhancement and subarachnoid involvement often lead to increased intracranial pressure symptoms such as headache, nausea, papilledema, and cranial nerve issues, indicating meningeal irritation and CSF flow problems. Focal parenchymal masses, such as chloromas, visible on MRI or CT scans, are associated with localized neurological deficits, seizures, and symptoms from mass effect [36,84]. Additionally, nerve root or optic nerve enhancement may correlate with radiculopathy and visual impairments [36]. Notably unlike ALL, where CNS prophylaxis with intrathecal chemotherapy is essential due to high CNS relapse risk, routine CNS prophylaxis and imaging are not recommended in adult AML because of its lower CNS involvement and lack of evidence for benefit. Neuroimaging is typically for patients with neurological symptoms or suspicion, while systemic therapies like cytarabine may incidentally protect against CNS relapse [99]. However, CNS evaluation may be considered for high-risk patients, and more research is needed to determine optimal early detection and prevention strategies.

Several challenges and limitations affect the utility of neuroimaging for detecting CNS involvement in patients with AML. A recent study by Galldiks et al. highlights key limitations of advanced MRI in brain tumors, such as ADC mapping, which is often interpreted visually and may be misleading in lesions surrounded by edema [100]. The same study also states that CBF estimation with arterial spin labeling (ASL) depends on blood velocity and selecting an appropriate post-labeling delay, which can vary across patients and with age [100,101]. Standard MRI sequences have also limitations, particularly in distinguishing intracerebral tumors—such as chloromas in AML—from metastases, infections, or abscesses [102]. Conventional sequences are also insufficient for reliably assessing tumor cellularity or grade, necessitating more specific or invasive methods, such as CSF cytology, to evaluate these characteristics [14,103]. Furthermore, neuroimaging findings may also influence therapeutic decision-making and treatment monitoring in AML patients with CNS involvement. The detection of parenchymal chloromas or leptomeningeal disease on MRI often prompts the initiation of CNS-directed therapy, including intrathecal chemotherapy and intensified systemic regimens. Serial imaging can be used to assess treatment response, with reductions in lesion size, contrast enhancement, and diffusion restriction reflecting therapeutic efficacy [104]. Also, neuroimaging plays a critical role in identifying chemotherapy-related complications, such as intracranial hemorrhage, ischemic injury, or toxic leukoencephalopathy, which may necessitate treatment modification [94,99]. Thus, imaging serves as an essential adjunct in tailoring and monitoring chemotherapy in AML-related CNS disease.

Overall, each imaging modality provides complementary insights into CNS involvement in AML. CT is mainly employed in emergency situations to quickly identify intracranial hemorrhage, mass effect, or hydrocephalus, but has limited ability to detect subtle parenchymal or leptomeningeal abnormalities. MRI is considered the gold standard for CNS assessment due to its superior soft-tissue contrast and ability to identify parenchymal infiltration, meningeal enhancement, lesion cellularity as assessed by DWI, and treatment-related changes [91,96,104]. PET, usually performed alongside CT, is a useful additional tool for detecting metabolically active extramedullary disease and monitoring treatment response, although its sensitivity for leptomeningeal disease is limited. Therefore, a multimodal imaging approach is often necessary to effectively detect and characterize CNS disease in AML.

**Table 2:** Main neuroimaging features appearing in AML patients with CNS infiltration.

Study [Ref]	Pathologic Subtype/Type of Involvement	Imaging Modality	Main Radiologic Findings	Location
Direct CNS Infiltration				
Akhaddar et al., 2011 [51] Woo et al., 1986 [84] Guermazi et al., 2002 [85]	Chloroma/Myeloid Sarcoma	CT	Isodense or hyperdense soft-tissue masses. Multiple small lesions more common than solitary large masses; minimal contrast enhancement; possible surrounding hypodense edema.	Parenchymal: may show peripheral enhancing rim mimicking abscess. Extraaxial/dural-based: Mimic meningiomas or leptomeningeal metastases; typically lack calcifications and may cause bone destruction Neuro-ophthalmic infiltration Spinal cord/cauda equina
Guermazi et al., 2002 [85] Hakyemez et al., 2007 [86] Nabavizadeh et al., 2016 [87] Ooi et al., 2001 [88]		MRI (T1WI)	Iso- or hypointense on T1WI with homogeneous contrast enhancement; perilesional hypointense edema and mass effect.	
		MRI (T2WI & FLAIR)	Hyper- or isointense tumors on T2WI and FLAIR.	
Hakyemez et al., 2007 [86] Nabavizadeh et al., 2016 [87]		MRI (ADC maps)	Restricted diffusion due to high tumor cellularity appearing hypointense.	
Verger et al., 2022 [90] Karlin et al., 2006 [91]		PET/CT	Focal radiotracer uptake in meningeal or intracerebral sites; useful for identifying extramedullary disease in AML patients.	
Nguyen et al., 2023 [50] Gleissner et al. 2006 [92] Collie et al., 1999 [93] Liu et al., 2017 [94]	Leptomeningeal disease	MRI (T1WI)	Pial surface or nodular leptomeningeal enhancement; often multifocal Meningeal (commonly dural) thickening	May involve cranial/spinal nerves, ventricular ependyma, tentorium, or cerebral convexities.
Indirect CNS Infiltration				
Algharras et al., 2013 [96] Nabavizadeh et al., 2016 [87] Liu et al., 2016 [73]	Leukostasis-re lated infarcts/hemorrhage	CT	Hyperdense intracerebral lesions resembling chloromas (hemorrhage)	Intracerebral cortex Peri-ventricular white matter Basal ganglia
		MRI (SWI)	Low signal and blooming artifacts representing chronic blood products and microbleeds.	
		MRI (DWI)	Hyperintense foci in periventricular white matter or consistent with ischemic lesions.	
Castro et al., 2025 [97]	Hypoxic injury	CT/MRI	Hypodense on CT, hyperintense on T2WI/FLAIR with restricted diffusion and mild enhancement.	

### 3.4. Artificial Intelligence and Computational Imaging

AI has noticeably elevated the use of neuroimaging modalities. Initially, machine learning (ML) has been used in neuroimaging to detect MRI patterns by training models on labeled (supervised learning) or unlabeled (unsupervised learning) data, enabling the prediction of previously unseen information and the detection of specific patterns [105]. Analogous to supervised systems, reinforcement learning is initially trained with labeled data, after which unlabeled data can further enhance the model’s classification performance [106]. Recent progress in AI, Deep learning (DL), is a subset of ML that uses artificial neural networks (ANNs) with multiple layers to automatically learn patterns from large and complex datasets [21]. Various AI models have been developed to assist in AML sub-classification, prognosis prediction and guide therapeutic decisions, thereby enabling tailored treatment by integrating clinical and molecular data [107,108,109,110].

In neuroimaging, ML and DL techniques are applied across multiple critical stages of MRI analysis, beginning with preprocessing steps such as skull stripping and bias-field correction, and extending to image segmentation, pattern recognition, disease detection, classification, and ultimately prognosis prediction based on imaging biomarkers [106,111]. Models specifically developed for CNS disease manifestations due to AML are limited; therefore, relevant studies from recent years investigating brain metastases’ detection and prognosis will be presented. A study by Zeeshan A. et al. [22] introduced AML-Net, an attention-based multi-scale lightweight model for brain tumor segmentation in MRI. AML-Net achieved superior performance (IoU = 0.834, F1 = 0.909, sensitivity = 0.939) while using significantly fewer parameters than existing models, demonstrating improved efficiency and accuracy for clinical brain MRI segmentation [22]. A different research presented an automated 3D template-matching algorithm to detect brain metastases (including chloromas) on conventional MRI scans. By employing spherical tumor models and 3D normalized cross-correlation in the frequency domain, the method efficiently identified likely lesion sites, achieving 89.9% sensitivity [23]. A retrospective study developed a DL-based computer-aided detection (CAD) system for automated detection and treatment response evaluation of brain metastases on MRI. CAD achieved sensitivities ranging from 75.1% to 94.7%, with mean Dice similarity coefficients up to 0.82 and low false-positive rates [24]. A recent DL-based convolutional neural network (CNN) model, Brainlab Smart Brush, was evaluated for automated detection and segmentation of brain metastases on MRIs, demonstrating high volumetric agreement with manual segmentation (ρ = 0.997, *p* < 0.001) and superior sensitivity for lesions >0.1 cc (97.5% vs. 93%), highlighting its potential to enhance accuracy and efficiency in neuro-oncology workflows [25]. Within the same spectrum, AI-based models could assist in detecting myeloid sarcomas that affect the CNS, characterize their imaging features, and thereby contribute to prognostic assessment in affected patients.

Radiomics is an emerging field in neuroimaging that seeks to uncover hidden patterns in both common and rare medical conditions. Like other -omics disciplines (such as genomics, proteomics, and metabolomics), radiomics extracts subtle imaging features and fine intensity variations from large-scale medical data that may be overlooked by the human eye [112,113]. By quantifying these imperceptible image characteristics in CNS leukemia and analyzing lesion heterogeneity, radiomics aims to associate these features with diagnostic, prognostic, and predictive outcomes, while also enabling the prediction of tumor response—such as chloromas—to chemotherapeutic agents and the potential resistance of malignant cells to specific treatment options [112,114]. Several studies have explored the potential of radiomics in differentiating glioblastoma, a high-grade glioma with poor prognosis, from primary central nervous system lymphomas using ML or DL approaches [115,116,117], with radiomic analyses of CNS involvement in myeloid leukemia remaining scarce due to the condition’s rarity. Radiomics is also crucial for the characterization of brain metastases in neuro-oncology. A relevant case is the 2025 study by Wu K. C. et al., which developed and evaluated the Adaptive RAG Assistant MRI Platform (ARAMP), an AI-based system integrating radiomic analysis with a large language model for brain metastasis detection on post-contrast T1WI MRI. ARAMP improved diagnostic sensitivity from 0.84 to 0.98 and enhanced inference consistency among neuroradiologists [26]. The results suggest that adaptive RAG-based AI frameworks can improve diagnostic accuracy and standardization in neuroimaging workflows, thereby strengthening the disease detection and risk characterization of less common cases, such as parenchymal myeloid sarcomas in AML patients.

Various clinical entities exhibit CNS imaging features similar to those observed in AML-related disease. A recent trend in AI applications in neuroimaging involves using transfer learning (TL) methods, which enable DL or ML models trained on common pathologies, such as meningiomas, to be adapted for rarer conditions, such as AML-related CNS involvement. TL leverages the extensive availability of training datasets for well-studied disease ontologies to enhance the detection and classification performance of models applied to rarer conditions, which typically lack large, curated image libraries and annotated datasets [118]. An example of TL application is the development of a deep TL-radiomics (DTLR) nomogram for meningioma grade prediction, which combines clinical, radiomic, and DTL features, achieving an AUC of 86.6% [27]. A different approach implicating TL in meningioma grading involves using a pre-trained CNN with data augmentation based on ImageNet-trained VGG-19. This model achieved up to 98.9% accuracy in distinguishing low- to high-grade tumors [28]. Initially developed for meningioma grading, these models could similarly be adapted to AML-related CNS disease, supporting improved lesion detection, classification, and prognostic assessment in this rare condition. In this context, fine-tuning pre-trained models on smaller, disease-specific datasets could further optimize their performance for identifying AML-related CNS manifestations, even when only limited annotated data are available, as applied in the Talukder M. A. study on brain tumors [29].

Some attempts regarding leukemia diagnosis have employed TL-based models to improve classification accuracy from microscopic blood smear images. By integrating advanced image processing, data augmentation, and deep CNNs such as Inception-ResNet, these approaches achieved F1 scores above 95% [30]. Analogously, this could be applied in neuroimaging detection of CNS involvement in AML, with clinical entities that resemble leptomeningeal enhancement and myeloid sarcomas observed in AML. With the guidance of the 2024 Artificial Intelligence in Response Assessment in Neuro-Oncology (AI-RANO) recommendations for standardization and validation, AI applications in CNS tumor imaging can be further leveraged through TL approaches to improve the detection and characterization of CNS involvement in AML [119,120]. For instance, parenchymal or extra-axial chloromas can mimic meningiomas or other primary CNS tumors, presenting as dural-based, homogeneously enhancing extra-axial masses on T1WI, with similar MRI signal characteristics and nonspecific neurological symptoms [121,122]. AI models developed for detecting leptomeningeal metastases from solid tumors could also be fine-tuned and repurposed to identify leptomeningeal enhancement associated with AML-related CNS disease [50].

**Figure 3:**
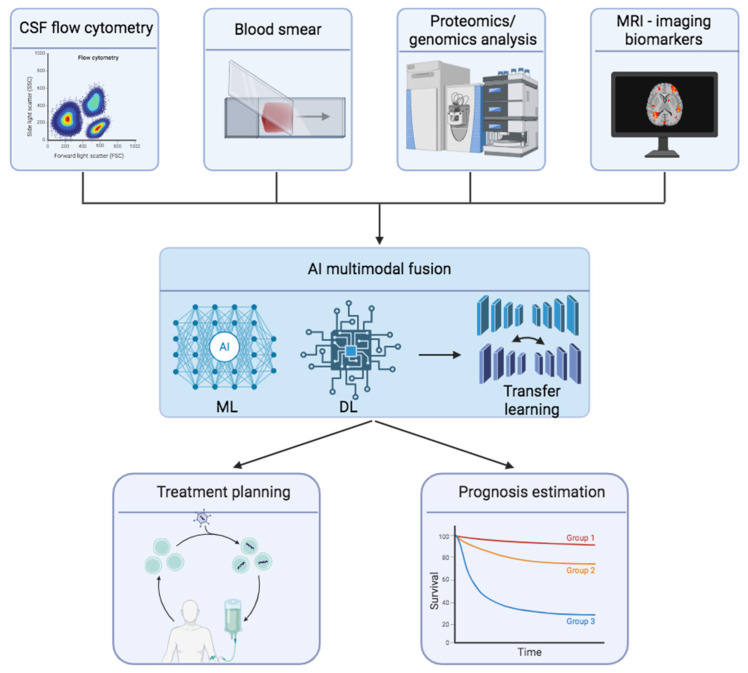
Data from multiple diagnostic sources are integrated through artificial intelligence-based multimodal fusion. Machine learning (ML), deep learning (DL), and transfer learning approaches combine these heterogeneous inputs to enhance diagnostic precision, support individualized treatment planning, and enable prognosis estimation in AML patients with CNS involvement. Created in BioRender. Christodoulou, R. (2026) Available online: https://BioRender.com/9pcaqgz (accessed on 20 December 2025).

Multimodal data fusion could further enhance the detection of CNS involvement in AML, as patients often present with diverse symptomatology arising from multiple extramedullary sites (Figure 3). Integrating different diagnostic methodologies, such as CSF and hematologic biomarkers, could improve overall diagnostic accuracy and sensitivity [123]. A recent study applied multi-omics integration to classify AML patients into molecular clusters, identifying FLT3, WT1, and KIT mutations associated with the best prognosis, while RUNX1, DNMT3A, and TP53 mutations were associated with poor outcomes [124]. Another study focused on oxidative stress-related prognostic genes in AML by developing a model that evaluated clinical outcomes and chemotherapeutic sensitivity, signaling pathway impacts, and correlations with immune scores and dysfunction, aiming to identify genes as potential prognostic biomarkers [125]. While these multi-omic and molecular approaches show promise, MRI-based radiomics for CNS disease has yet to be widely applied, mainly due to limited imaging datasets and the rarity of such cases.

### 3.5. Current Datasets and Data Challenges

The primary limitation in applying AI for the neuroimaging detection of CNS involvement in AML is the limited availability of publicly accessible imaging datasets. This constraint significantly hampers the development and validation of reliable ML and DL models, which require large, diverse datasets to achieve robust, reproducible performance [126]. TL and fine-tuning strategies can be applied to mitigate this limitation, allowing models initially trained on larger, well-annotated pre-existing datasets to be adapted for rarer pathologies such as AML-related CNS disease [127].

Some potential surrogate datasets for model pretraining include public MRI repositories of brain metastases or primary brain tumors, where MR scans and additional radiomics analyses can be conducted to characterize lesions, providing insights into their key features [128]. Such datasets include the University of California San Francisco Preoperative Diffuse Glioma MRI (UCSF-PDGM) dataset, which provides for approximately 500 patients suffering from diffuse glioma [129], and the Yale New Haven Hospital (YNHH) dataset containing 200 patients who have been diagnosed with brain metastases and underwent MRI scan for T1WI post-contrast, T2WI, and FLAIR sequences [130]. A large open-source dataset that could support AI model training and fine-tuning is the Multimodal Brain Tumor Image Segmentation Benchmark (BraTS) Pre-operative Meningioma Dataset, which includes over 1000 publicly available MRI scans and corresponding annotations [131]. This dataset could be harnessed for modeling leptomeningeal disease enhancement, which in AML can exhibit imaging features similar to those seen in specific meningioma cases. Additional neuroimaging resources, such as OpenNeuro [132] and the Cancer Imaging Archive (TCIA) [133], can also be leveraged for model initialization before domain-specific fine-tuning on smaller AML datasets.

Given the rare incidence of CNS involvement in acute myeloid leukemia, assembling open-source large datasets across institutions is essential for developing and fine-tuning reliable AI models. However, data usage is often restricted by privacy regulations and ethical considerations [134]. In this context, federated learning and privacy-preserving AI frameworks offer promising solutions. These approaches enable collaborative model training across multiple institutions without exchanging raw patient data, thereby maintaining compliance with data protection standards, such as HIPAA in the US and GDPR in Europe [135]. By allowing models to learn from distributed and heterogeneous datasets, federated AI enhances generalizability, reduces bias, and promotes reproducibility while safeguarding patient confidentiality.

### 3.6. Clinical Applications and Future Directions

AI-assisted neuroimaging diagnosis could significantly improve CNS disease detection in AML [19]. Due to the rarity of the manifestations, clinicians often do not initially suspect CNS involvement in leukemic patients, leading to the oversight of subtle or nonspecific symptoms. However, establishing a standardized diagnostic protocol comprising a defined set of examinations and non-invasive procedures—such as neuroimaging with CT and MRI—could enable earlier detection of CNS disease before the onset of severe neurological complications [136]. ML and DL models, supported by radiomic feature extraction, could substantially improve disease diagnosis, as many CNS manifestations present with subtle imaging findings that are difficult to discern through visual assessment alone. For instance, leptomeningeal infiltration may appear merely as mild dural thickening and faint hyperintense signals, which can easily be overlooked in conventional radiological evaluation [50]. Several AI models have also been developed and utilized for disease prediction. An example of this could be predicting CNS relapse risk from baseline imaging and hematological data for AML patients. Technologies predicting the risk of recurrence were trained on multiple clinical and molecular features, demonstrating high performance metrics and potential clinical applicability. For instance, a recent bioinformatics study leveraging expression data of 226 PANoptosis-related genes identified two distinct molecular clusters with unique immune profiles and survival outcomes, constructing a 19-gene prognostic signature capable of accurately stratifying AML patients into high- and low-risk groups [137]. This can similarly be applied to risk prediction in patients with CNS involvement in AML, based on radiomics signatures, or harnessing multimodal fusion.

Future directions in the study and management of CNS involvement in AML focus on improving early detection, diagnostic precision, and personalized therapy. AI-based predictive models integrating baseline imaging, genomic, and hematologic data could help identify patients at risk of CNS relapse. At the same time, liquid biopsy and CSF molecular profiling offer minimally invasive tools for early detection [123]. Integrating multimodal imaging (MRI, PET, CT) and multi-omics data can provide a comprehensive understanding of disease biology and the mechanisms of CNS invasion, including the role of adhesion molecules, cytokine expression, and microenvironmental interactions with the blood–brain barrier [138]. Furthermore, establishing large, federated, and privacy-preserving databases is essential for robust model training and external validation, enabling reproducible AI research while safeguarding patient confidentiality [135]. Finally, longitudinal monitoring combining serial imaging, CSF biomarkers, and AI-assisted analysis may allow real-time disease progression and relapse risk assessment, paving the way toward precision neuro-oncology in AML [124].

## 4. Conclusions

CNS involvement in AML is rare but significant, often presenting subtly with myeloid sarcoma or leptomeningeal enhancement. Advances in MRI have improved detection, but limited research and datasets hinder the development of diagnostic criteria. AI and radiomics could improve diagnosis, risk stratification, and prognosis, mainly via transfer learning and model tuning, but their potential is limited by data scarcity. Future efforts should focus on multicenter collaborations, federated learning, and the integration of imaging, molecular, and clinical data to enable personalized diagnosis and treatment for this rare but impactful disease.

## Figures and Tables

**Table 1:** Artificial Intelligence and Computational Imaging Studies Relevant to CNS Imaging and AML-Related Applications.

Study [Ref]	Clinical Context	Imaging Modality	AI/Computational Method	Task	Key Performance Metrics
Zeeshan et al., 2024 [22]	Brain tumors (applicable framework for CNS myeloid sarcoma)	MRI	Attention-based multi-scale CNN (AML-Net)	Tumor segmentation	IoU = 0.834; F1-score = 0.909; Sensitivity = 0.939
Ambrosini et al., 2010 [23]	Brain metastases (including hematologic lesions)	MRI	Automated 3D template matching with normalized cross-correlation	Lesion detection	Sensitivity = 89.9%
Cho et al., 2021 [24]	Brain metastases	MRI	Deep learning CAD system	Detection and treatment response assessment	Sensitivity = 75.1–94.7%; Dice coefficient up to 0.82
Madhugiri et al. 2025 [25]	Brain metastases	MRI	CNN-based automated segmentation	Detection and volumetric segmentation	Volumetric agreement ρ = 0.997 (*p* < 0.001); Sensitivity 97.5% for lesions >0.1 cc
Wu et al., 2025 [26]	Brain metastases	Post-contrast T1WI MRI	Radiomics + RAG-assisted large language model	Lesion detection and reporting	Sensitivity improved from 0.84 → 0.98; improved inter-reader consistency
Li et al., 2025 [27]	Meningioma (transfer learning model relevant to AML mimics)	Multiparametric MRI	Deep transfer learning radiomics nomogram	Tumor grading	AUC = 0.866
Fasihi Shirehjini et al., 2025 [28]	Meningioma	MRI	Transfer learning CNN (ImageNet-pretrained VGG-19)	Tumor grade classification	Accuracy up to 98.9%
Talukder et al., 2023 [29]	Brain tumors	MRI	Fine-tuned CNN with reconstruction-based learning	Tumor classification	Accuracy > 95%
Haque et al., 2024 [30]	Leukemia diagnosis (non-imaging CNS analogue)	Microscopy images	Transfer learning CNN (Inception-ResNet)	Leukemia classification	F1-score > 95%

## Data Availability

No new data were generated.

## Abbreviations

The following abbreviations are used in this manuscript:

ADC	Apparent Diffusion Coefficient
AI	Artificial Intelligence
AML	Acute Myelogenous Leukemia
APL	Acute Promyelocytic Leukemia
AUC	Area Under the Curve
BMs	Brain Metastases
CNS	Central Nervous System
CSF	Cerebrospinal Fluid
CT	Computed Tomography
CNN	Convolutional Neural Network
DL	Deep Learning
DTLR	Deep Transfer Learning Radiomics
DWI	Diffusion-Weighted Imaging
EEG	Electroencephalography
fMRI	Functional Magnetic Resonance Imaging
FLAIR	Fluid-Attenuated Inversion Recovery
ITD	Internal Tandem Duplication
ML	Machine Learning
MRI	Magnetic Resonance Imaging
PET	Positron Emission Tomography
SWI	Susceptibility-Weighted Imaging
TCIA	The Cancer Imaging Archive
TL	Transfer Learning
T1WI/T2WI	T1-Weighted/T2-Weighted Imaging
WBC	White Blood Cell
